# Arginine accumulation suppresses heat production during fermentation of the biocontrol fungus *Beauveria bassiana*

**DOI:** 10.1128/aem.02134-24

**Published:** 2025-02-05

**Authors:** Tong Wu, Fangfang Zhan, Liqiong Zeng, Yanli Sun, Shihui Fu, Yu Fang, Xiaochun Lin, Haoyu Lin, Jun Su, Shouping Cai

**Affiliations:** 1 Fujian Provincial Key Laboratory of Haixia Applied Plant Systems Biology, Haixia Institute of Science and Technology, Fujian Agriculture and Forestry University 12449 , Fuzhou, China; 2 Fujian Academy of Forestry, Key Laboratory of National Forestry and Grassland Administration on Timber Forest Breeding and Cultivation for Mountainous Areas in Southern China 425050 , Fuzhou, China; 3 Forestry Development Center of Lanshan District in Linyi, Shandong, China; 4 Liancheng Forestry Bureau, Liancheng, China; 5 Institute of Resources, Environment and Soil Fertilizer, Fujian Academy of Agricultural Sciences 107629 , Fuzhou, China; 6 Fuding Forestry Bureau, Fuding, China; Royal Botanic Gardens, Surrey, United Kingdom

**Keywords:** *Beauveria bassiana*, solid-state fermentation, multiomics, arginine, heating period

## Abstract

*Beauveria bassiana* (*Bb*) is one of the most widely used biocontrol agents, and its products constitute more than one-third of the global market share of fungal insecticides. Solid-state fermentation (SSF) is widely used in the production of *Beauveria bassiana* (*Bb*) because of its economic practicality and high production efficiency. However, the heat generated during fermentation can sharply reduce both the yield and quality of *Bb*, and current industrial methods to mitigate high temperatures during fermentation are inadequate, leading to increased production costs. Thus, exploring the underlying mechanism of how heat is produced by *Bb* is crucial for improving the SSF procedure and yield. This study employed multiomics data analysis of *Bb* during SSF to explore the relationships between fungal fermentation and environmental factors. We found that the heat production period for SSF was 12 hours to 48 hours post-inoculation. To further explore the underlying mechanism during this heating period, we identified 454 temperature-correlated metabolites (TCMs) and 1,994 temperature-correlated genes (TCGs). Annotations of the above TCMs and TCGs revealed significant enrichment in the arginine biosynthesis pathway; specifically, the expression level of glutamine synthetase, a TCG, decreased with fermentation time, whereas the expression levels of the TCGs L-arginine and L–glutamine increased with fermentation time, and glutamine synthetase and L-glutamine in the arginine biosynthesis pathway cycle produced the end product L-arginine. Furthermore, when the substrates of the SSF were treated with exogenous arginine, the temperature peak of the SSF significantly decreased with increasing concentration of exogenously added arginine.

**IMPORTANCE:**

A large amount of experimental evidence from the field has shown that *Bb* is an irreplaceable mature product that protects the health of our agriculture and ecosystem. In addition to high efficiency and host extensiveness, low cost is a critical merit that makes *Bb* products frequently used in the field. However, the growing cost of power and labor in the *Bb* industry, especially the SSF procedure, has significantly increased the price of its products, thus restricting the use of *Bb* in the field. This study not only fills the theoretical knowledge gaps concerning the molecular basis of the interrelationship between *Bb* and the fermentation environment during SSF but also provides an economical and applicable strategy (the addition of arginine to the fermentation media) to further lower the cost and increase the yield of *Bb* during SSF at the industrial level.

## INTRODUCTION

*Beauveria bassiana* has been approved as a safe biopesticide for agricultural purposes; it can control more than a dozen pests and is widely used worldwide (1–5). *Bb* can be produced via different methods. Spores are the main application form of *Bb*, and the main method of obtaining spores is solid-state fermentation (SSF). SSF is considered the most efficient and widely used method for large-scale production (6). It requires less capital, energy, and labor because it does not require strict control of the fermentation parameters (7). In the initial stages of SSF, substrates are metabolically broken down into components that are more conducive to the nutritional growth of *Bb*. During this process, the temperature and CO_2_ level increase (8). However, excessive CO_2_ accumulation can reduce the yield of asexual spores (9), and metabolic heat generated by fermentation substrates can lead to contamination by unwanted microorganisms (8, 10). Currently, there are no reports on the metabolic pathways of *Bb* during SSF. Understanding these pathways provides a theoretical basis for selecting more suitable substrates and regulating environmental factors during cultivation in the future. If the fermentation process were to be improved by precisely controlling the environmental conditions, the cost investment would increase, which would make it unsuitable for industrial upgrading (7). Therefore, a new method to improve the current fermentation process is urgently needed.

In SSF, the fermentation temperature increases gradually due to metabolic heat production, reaching a maximum and then slowly decreasing to ambient temperature (11, 12). A large quantity of metabolic heat is produced during SSF, reaching 3,200 kcal.kg^−1^ dry matter in composting systems and a temperature gradient of 3°C.cm^−1^ during the fermentation of tempeh (13). In the fermentation medium of SSF, microbiome-derived organic matter (such as nutrients and enzymes) and factors affecting pH are produced, creating a heterogeneous environment for fungal metabolism (8), which affects the colonization and sporulation of *Bb* (14). However, if the metabolic heat is not removed in time, it can also affect the growth of the fungi. Heat generation can cause water evaporation, leading to substrate drying, which affects spore formation and product formation (13).

Therefore, heat generation is undesirable in SSF. However, in SSF, the effective thermal conductivity of porous media, which are usually made of organic materials, is poor, which hinders heat dissipation (13). Currently, there are many examples of improving heat dissipation by modifying fermentation reactions, such as by using air conditioning to control the temperature (15), using stirred tank reactors instead of tray reactors (16), and expanding internal air circulation in fermentation chambers (16). However, these improvements do not fundamentally solve the need for heat dissipation and include additional costs. If the heat dissipation problem can be addressed by improving the raw fermentation materials or strains, the SSF of *Bb* can be fundamentally optimized. There are many unknowns regarding the biochemical basis of the increased temperature stage in the SSF of *Bb*, and further exploration of this process is critical for optimizing it. In the SSF of *Bb*, the increase in temperature is an important factor affecting spore formation and spore quality. To expand the production of *Bb*, a deep understanding of the increase in temperature and the mechanisms influencing it is necessary.

*Bb* obtains nutrients by decomposing the culture medium (13), and clarifying the metabolites produced from this decomposition is crucial for understanding the fermentation process. The widespread use of transcriptomics and metabolomics analysis technologies has revealed the metabolic processes and gene expression mechanisms involved in various microbial fermentation processes (17–20). Numerous similar studies have demonstrated that the combined analysis of transcriptomics and metabolomics can establish logical relationships among genes, metabolites, and phenotypes, thereby elucidating the changes in microbes during fermentation processes and their interactions with culture media or fermentation environments. However, owing to the lack of such studies, research on the solid-state physiology and biochemistry of *Bb* during fermentation is severely limited, hindering the improvement and optimization of the fermentation process.

In this study, we first established a correlation between the growth of *Bb* and the fermentation environment by observing the process of fermenting *Bb*. Through subsequent multiomics analysis (transcriptome and metabolome) of *Bb* and culture media mixtures at different fermentation times and correlation analysis of the data from the two omics approaches, we further explored the physiological and biochemical characteristics of *Bb* during fermentation. This work provides a theoretical basis for the standardization and optimization of the SSF of *Bb*.

## MATERIALS AND METHODS

### Solid fermentation of *Beauveria bassiana*

The *Beauveria bassiana* strain (BbFZ-51) used in this study was isolated and preserved by the Fujian Academy of Forestry Sciences, Fuzhou. This strain has been patented by the China General Microbiological Culture Collection Center (CGMCC NO.41505). The *Bb* mycelia were incubated for 14 days at 25±1°C with a 12 L:12 D photoperiod (SPX - 300BSH - II, Shanghai Xinmiao Experimental Equipment Co., Ltd., Shanghai, China) on potato dextrose agar (PDA) media. The spore powder was then scraped into soybean flour medium (liquid medium consisting of 2% sucrose +4% soybean powder) and incubated for 3 days at 25 ± 1°C with a 12 L:12 D photoperiod (SPH - 211C, Shanghai Shiping Experimental Equipment Co., Ltd., Shanghai, China). After a large volume of mycelial liquid was obtained, *Bb* was incubated in a 30-L liquid fermenter (RTY-MZ, Zhenjiang Ritai Biological Engineering Equipment Co., Ltd., Zhenjiang, China). The culture conditions were as follows: temperature, 25 ± 1°C; time, 48 hours; photoperiod, 24 D; and medium, 2% sucrose +4% soybean powder. The mycelial mixture was subsequently inoculated onto solid medium (wheat bran: rice husk: water at a weight ratio of 4:1:4) at a rate of 20% (wt/vol), and solid fermentation commenced. The culture conditions were as follows: temperature, 22 ± 1°C; duration, 10 days; and photoperiod, 24 D. Samples were taken at 12 hours, 24 hours, 30 hours, 48 hours, 60 hours, and 96 hours, with three biological replicates at each time point (50 g per biological replicate). The samples were rapidly frozen in liquid nitrogen and stored at −80°C for subsequent experiments.

### Quantification of temperature and CO_2_ during solid fermentation

The solid fermentation chamber for *Bb* was developed by the Fujian Academy of Forestry Sciences, Fuzhou (21). Three CO_2_ concentration sensors (FD/RS-CO2WS-NO1-2-10000p, Shandong Renke Measurement and Control Technology Co., Ltd., China) and three temperature sensors (BC2385-ND, DigiKey, American) were placed on the upper, middle, and lower layers of the fermentation chamber (located in the 10th, 6th, and 1st layers of the trays, respectively). Data were recorded every 30 minutes and automatically uploaded to the cloud platform (FujinCloud, Ningbo, China).

### Quantification of *Bb* mycelia

The samples at 12 hours, 24 hours, 30 hours, 48 hours, 60 hours, and 96 hours were ground with liquid nitrogen, total RNA was extracted with a Magen total RNA extraction kit (R4151-02, Magen, Guangzhou, China), and cDNA was synthesized from 1 mg of total RNA via cDNA synthesis supermix (CAT: 11141ES60, Yeasen, Shanghai, China) according to the manufacturer’s instructions. qRT‒PCR was performed using the cDNA template and gene-specific primer pairs (Table S1). qRT‒PCR analysis was performed with three biological replicates and three technical replicates with Hieff qPCR SYBR Green Master Mix (Low Rox Plus, part number 11202ES08, Yeasen, Shanghai, China) on a QuantStudio 6 Flex PCR system (ABI), and the results were analyzed via the 2^-Ct^ method. The data are expressed as means ± standard deviations (SDs) of six independent experiments.

### Quantification of spores

One gram of each sample was weighed at each time point (12 hours, 24 hours, 30 hours, 48 hours, 60 hours, and 96 hours), dispersed, and mixed with 4 mL of Tween-80 (CAS: 9005–65-6, Shanghai, China) by shaking. The spores were filtered with a lens paper, and the mixture was diluted 10 times. The spores were counted by optical microscopy (CX23LEDR88FS1C, Olympus Corporation, Japan) using a hemocytometer (02270113, Shanghai Precision Biochemical Instrument Co., Ltd., Shanghai, China). The hemocytometer had a size of 25 × 16, and the following formula was used for calculation: spore count/mL = spore count in 80 small squares/80 × 400 × 10^4^×dilution factor (22). Each sample was measured with three biological replicates and three technical replicates.

### Metabolomic analysis and data processing

The samples are mixtures of fungal media from each period (12 hours, 24 hours, 30 hours, and 48 hours). The metabolites were then extracted from each sample following a previously described protocol (23). Ultra-high-performance liquid chromatography (UHPLC) separation was carried out via a Dionex Ultimate 3000 RS UHPLC (Thermo Fisher Scientific, Waltham, MA, USA) instrument equipped with an ACQUITY UPLC HSS T3 column (1.8 µm, 2.1 × 100 mm, 186009468, Waters, Milford, USA) by Oebiotech Company (Shanghai, China). The flow rate was 0.35 mL/minute, and the mobile phases were 0.1% formic acid in water (A117-50, Thermo Fisher Scientific, Waltham, MA, USA) and 0.1% formic acid in acetonitrile (B) (A998-4, Thermo Fisher Scientific, Waltham, MA, USA). The column temperature was set to 45°C, the autosampler temperature was set to 4°C, and the injection volume was 5 µL (24, 25).

SCIEX Analyst Workstation Software (Version 1.6.3) was employed for MRM data acquisition and processing. MS raw data (.wiff) files were converted to the .txt format via msConvert. The R program and database were used for peak detection and annotation, respectively (26–28). Commercial databases, including the Kyoto Encyclopedia of Genes and Genomes (KEGG; http://www.kegg.jp) and MetaboAnalyst (https://www.metaboanalyst.ca/), were subsequently used to search for “metabolitepathways” (https://www.genome.jp/kegg/pathway.html).

### RNA-Seq and data analysis

Total RNA from samples was extracted via the TRIzol method (15596026, Invitrogen, CA, USA), and RNA purity was assessed via a NanoDrop spectrophotometer (IMPLEN, CA, USA). RNA integrity was evaluated via an Agilent 2100 Bioanalyzer (Agilent Technologies, CA, USA), and agarose gel electrophoresis was performed to assess RNA degradation. The extracted RNA was sent to Beijing Novogene Co., Ltd., for library construction and sequencing. After the cDNA libraries were constructed, the libraries were pooled, followed by sequencing on an Illumina HiSeq 4000 platform via a PE 150 sequencing strategy (library construction via reagent kits from Illumina, San Diego, USA). The *Beauveria bassiana* (BbFZ-51) genome was used as a reference for transcriptome analysis, and data from the genome were uploaded to the NCBI (GenBank accession number JBIQNX000000000). Raw data (raw reads) in fastq format were first processed through in-house Perl scripts, and cutadapt and Perl scripts were used to obtain clean data (29). All downstream analyses were based on clean, high-quality data. These clean reads were then mapped to the assembly, and annotation of the *Bb* reference genome was performed.

### Exogenous arginine application in solid fermentation of *Bb*

Solutions of arginine at concentrations of 1 g/L and 2 g/L (74–79-3, MACKLIN, Shanghai, China) were prepared, and the pH was adjusted to 6.0. The solid medium was prepared according to the method described in “Solid fermentation of *Beauveria bassiana*,” above, and three groups were established, each with three biological replicates. Arginine solutions (0 g/L, 1 g/L, and 2 g/L) were added to the three groups, which were subsequently fermented at a room temperature of 25 ± 1°C. The environmental factors were monitored via the equipment described in “Quantification of temperature and CO_2_ during solid fermentation,” above. During fermentation, samples were taken at 12 hours, 24 hours, 30 hours, and 48 hours; flash-frozen in liquid nitrogen, and stored in a −80°C freezer. The mycelia and spore contents were determined via the methods described in “Quantification of *Bb* mycelia” and “Quantification of spores,” above.

### Data analysis and visualization

GraphPad Prism 8 software was used to plot the data obtained for temperature, CO_2_, fungal mycelia, and spore content as line graphs. Pearson’s correlation analysis was performed to assess the correlations among the transcriptome, metabolome, and temperature, and correlation coefficients and *P* values were calculated. The website https://www.bioinformatics.com.cn was used to generate pie charts showing significantly correlated genes and metabolites. Significant metabolites were classified into secondary categories on the basis of the Human Metabolome Database (HMDB). Enrichment analysis of significant metabolites was performed via the KEGG metabolic pathway database. Data analysis and visualization were conducted via the OmicShare tool available at www.omicshare.com/tools.

## RESULTS

### The temperature increased from 12 hours to 48 hours during the SSF of *Bb*

**Fig 1:**
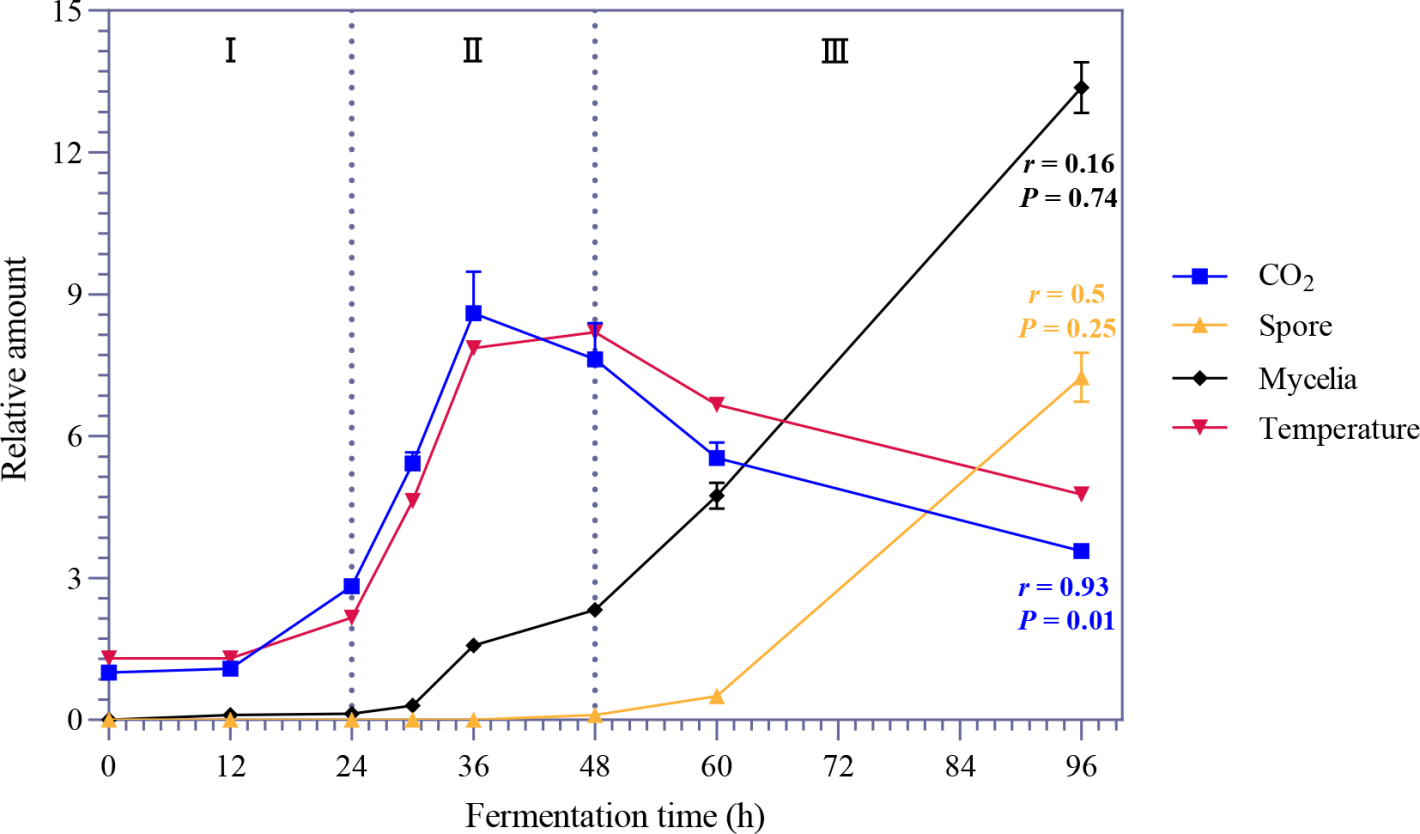
Dynamics of environmental factors and biomass during solid-state fermentation (SSF) of *Bb*. The first 96 hours of fermentation was assigned to three stages, Stage I (0–24 hours), Stage II (24–48 hours), and Stage III (48–96 hours), and the features of CO_2_, mycelia, spores, and temperature at different times were determined via fungal growth curves. All of the data are presented as means ± standard deviations (SDs). The *P* values represent the correlations between temperature and mycelia, spores, or CO_2_; on the basis of the Spearman correlation, a *P* value < 0.05 was considered statistically significant, and the strength of the correlation was calculated (**R**).

To clarify the molecular mechanism at work during the heating phase, we evaluated the temperature, CO_2_, and mycelia and spore contents. We found that between 12 hours and 96 hours during the SSF of *Bb*, the trends in CO_2_ and temperature were similar (*P* = 0.01). Both sharply increased from 24 hours onward and then slowly decreased from 48 hours onward. Mycelia began to grow slowly at 24 hours, and their growth increased dramatically from 48 hours onward, whereas the number of spores began to increase slowly at 24 hours (Fig. 1). On the basis of these four indicators, we divided the first 96 hours of SSF of *Bb* into three stages: the preparatory phase, lag phase, and logarithmic phase. The preparatory phase corresponds to the heating period, so we further studied the period from 12 hours to 48 hours.

### TCMs and TCGs are enriched mainly in the biosynthesis of various alkaloids, carbon metabolism, protein digestion, and absorption

**Fig 2:**
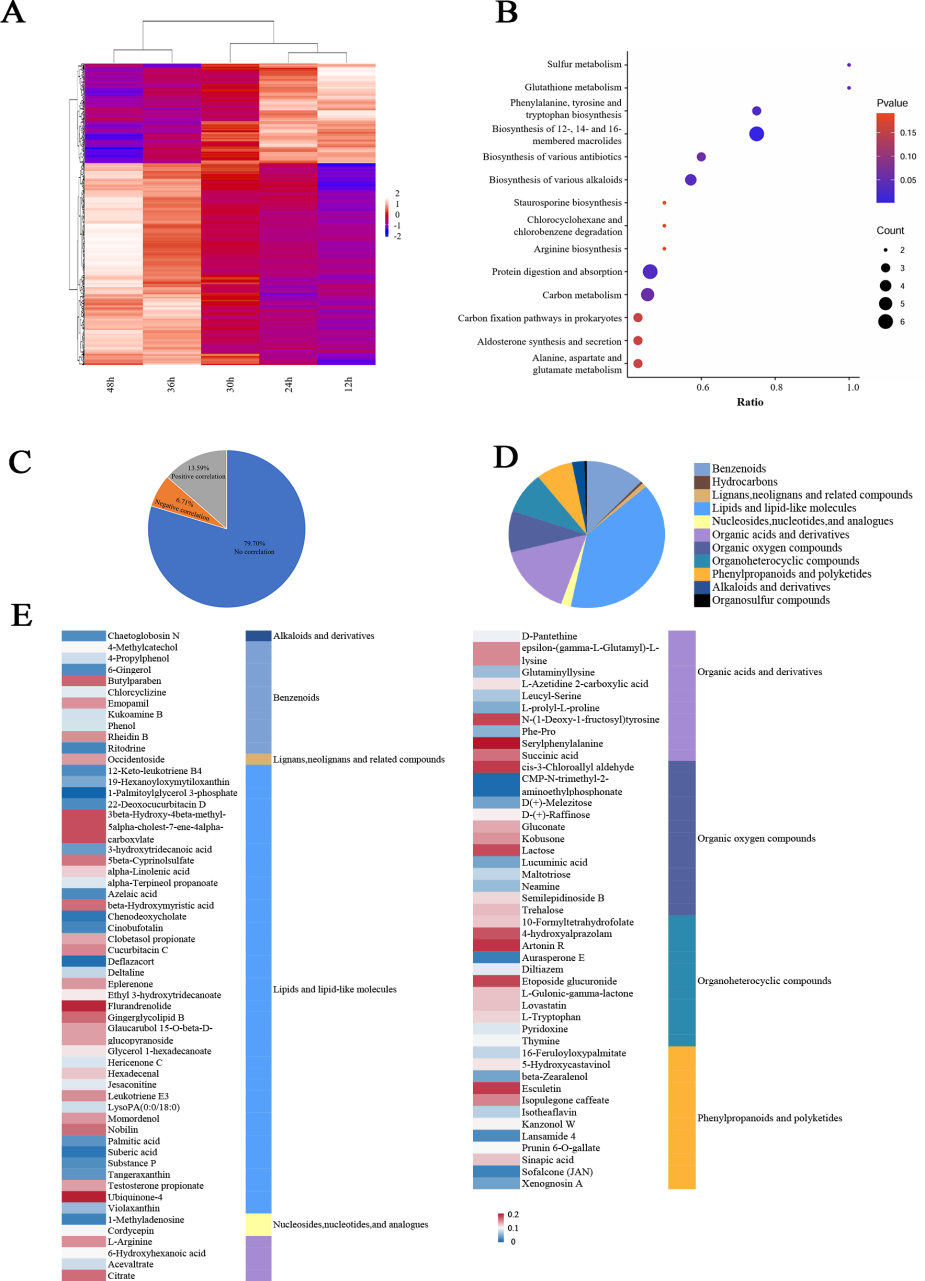
Temperature-correlated metabolites of *Bb* solid fermentation medium during Stages I–II. (**A**) Temperature-correlated metabolites (*P* < 0.05, based on the Pearson correlation) were plotted on a heatmap. The metabolite expression level was normalized by zero-mean normalization, from blue to red, and the higher the value was, the higher the expression level. (**B**) KEGG enrichment analysis of TCMs (temperature-correlated metabolites) is plotted, where the x-axis represents the proportion of TCMs in each pathway among all metabolites, and the y-axis represents the names of the identified pathways. The size of the circles indicates the number of significantly correlated metabolites within each metabolic pathway, and their coloring represents the *P* values for the enrichment analysis. (**C**) A pie chart displaying the number and proportion of TCMs (*P* < 0.05, based on the Pearson correlation). (**D, E**) Secondary classification of TCMs (*P* < 0.05, based on the Pearson correlation; secondary classification based on the HMDB). The metabolite expression level was normalized by zero-mean normalization, from blue to red, and the higher the value was, the higher the expression level.

RNA-seq and metabolomic analyses were conducted to explore the differences in *Bb* transcript levels and metabolites at different time points. In total, 43,588,801, 45,341,781, 40,035,122, 42,652,837, and 44,074,842 reads were sequenced from 12 hours to 48 hours. Correlation analysis between temperature changes from 12 hours to 48 hours and gene/metabolite abundance revealed significant correlations. At the transcriptome level, 23.88% of the genes were significantly correlated with temperature (*P* < 0.05), with 462 genes being positively correlated and 1,532 genes being negatively correlated (Fig. S2A and B). At the metabolome level, 20.30% of the metabolites were significantly correlated with temperature (*P* < 0.05), with 304 metabolites positively correlated and 150 metabolites negatively correlated (Fig. 2A and C). To further confirm the pathways associated with these metabolites and genes, we performed Kyoto Encyclopedia of Genes and Genomes (KEGG) enrichment analysis, which revealed enrichment of these correlated genes and metabolites in pathways such as carbon metabolism. TCMs are involved in carbon metabolism as carbon-containing metabolites or indirect metabolites. Further classification of the significant metabolites revealed a predominance of lipids and lipid-like molecules (alpha-linolenic acid, azelaic acid, etc.) and organic acids and derivatives (L-arginine, citrate, etc.) (Fig. 2D and E).

### Arginine is a crucial substrate for solid fermentation

**Fig 3:**
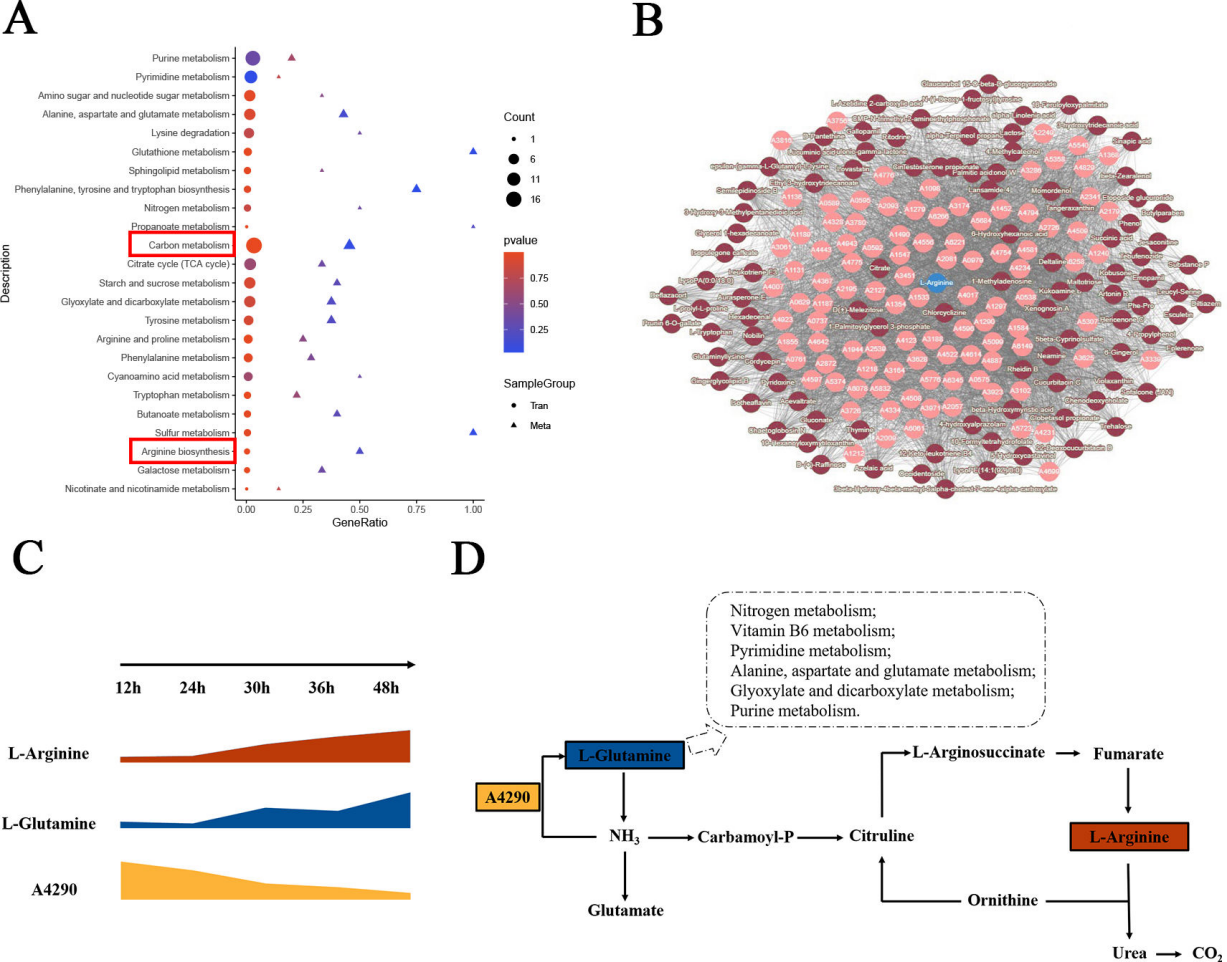
Arginine is a crucial substrate for SSF within Stages I–II. (**A**) KEGG enrichment analysis of TCGs (temperature-related genes) and TCMs enriched in the same pathway. The x-axis "Ratio" represents the ratio of the number of TCGs/TCMs in each KEGG pathway to all genes/metabolites. The y-axis represents the names of the pathways. Circles and triangles represent TCGs and TCMs, respectively, with the size indicating the number of TCGs/TCMs in that metabolic pathway, and the color represents the *P* value for the enrichment analysis. The most significantly enriched pathways (arginine biosynthesis and carbon metabolism) are highlighted. (**B**) Network diagram of TCGs and TCMs (*P* < 0.05, top 50 positively correlated genes, based on the Pearson correlation). Light pink nodes represent genes, whereas dark pink nodes represent metabolites. (**C**) Changes in the contents of TCGs and TCMs in the arginine biosynthesis pathway within Stages I–II. All of the data are presented as means ± standard deviations (SDs). (**D**) Arginine biosynthesis pathway. Boxes indicate TCGs/TCMs.

To more precisely target the pathways, we performed a joint analysis of genes and metabolites. Joint analysis of significant genes and metabolites revealed enrichment in different KEGG pathways, including carbon metabolism and arginine biosynthesis (Fig. 3A). Notably, carbon metabolism was enriched with 20 TCGs and five TCMs, whereas arginine biosynthesis was enriched with two TCGs and two TCMs. We also developed correlation network diagrams to further illustrate the relationships between significantly associated metabolites and significantly associated genes (Fig. 3B). L-arginine was significantly associated (*P* < 0.05) with the genes *MANBA* (beta-mannosidase), *HEXA_B* (hexosaminidase), and *DDI1* (DNA damage-inducible protein 1) (Table S2). On this basis, we further analyzed the main significantly related genes and metabolites involved in this pathway and their expression level changes (Fig. 3C and D). Within 12 hours to 48 hours, the levels of L-arginine and L-glutamine gradually increased, whereas the expression of the A4290 gene decreased progressively. Through the transcription and translation of the A4290 gene, L-glutamine is synthesized and enters arginine biosynthesis, eventually leading to the synthesis of L-arginine and the release of CO_2_, indicating that arginine biosynthesis directly affects the variation in CO_2_ content in the SSF of *Bb*. In addition, we analyzed the main significantly related genes and metabolites involved in the carbon metabolism pathway and their expression level changes (Fig. S3).

### Exogenous addition of arginine can increase the contents of spores and mycelia and affect the dynamics of environmental factors

**Fig 4:**
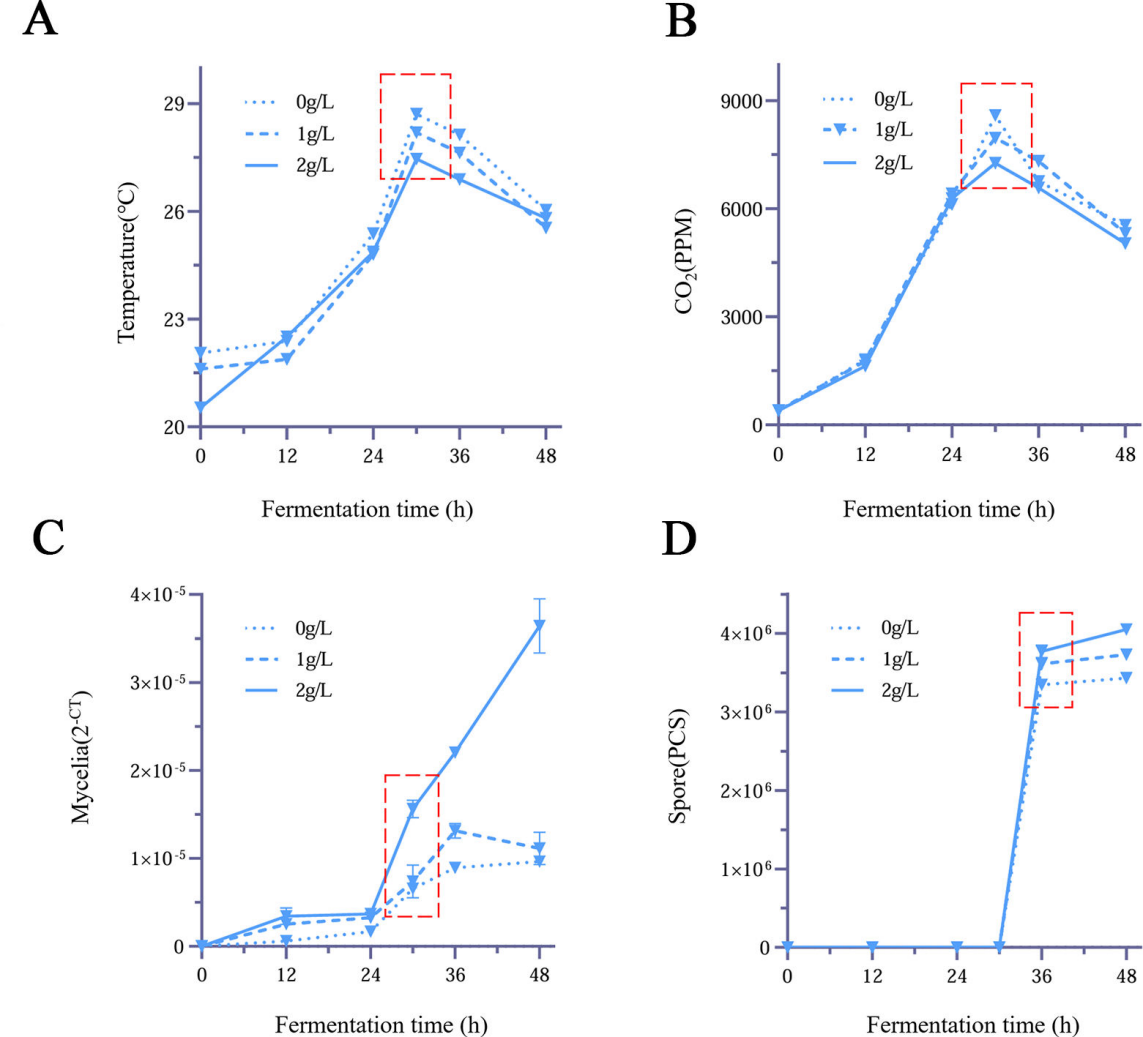
Impact of exogenous arginine application on the dynamics of environmental factors and biomass during the solid-state fermentation of *Bb* within Stages I–II. Arginine (0 g/L, 1 g/L, and 2 g/L) was exogenously added to the solid-state fermentation medium, and the changes in temperature (**A**), CO_2_ (**B**), mycelia (**C**), and spores (**D**) within Stages I–II were determined. The most obvious changes are highlighted. All of the data are presented as means ± standard deviations (SDs).

To investigate the function of arginine, we added different concentrations of arginine during SSF of *Bb* (Fig. 4). We found that after the addition of arginine, the contents of mycelia and spores increased (Fig. 4C and D), accompanied by decreases in fermentation temperature and CO_2_ content in the fermentation environment (Fig. 4A and B). These findings indicate that arginine can influence the SSF of *Bb*, increase the yield from *Bb*, and reduce the adverse effects of high temperature on the fermentation process. The changes caused by the addition of 2 g/L arginine were more significant than those caused by the addition of 1 g/L arginine.

### Metabolites are transmitted during SSF of *Bb* in stages I–II

We clustered the significantly related genes and metabolites from 12 hours to 48 hours (Fig. S4) and divided them into three stages, S1, S2, and S3, on the basis of the clustering results. To explore the relationships between significantly related genes and metabolites in different stages, we conducted Kyoto Encyclopedia of Genes and Genomes (KEGG) enrichment analysis. We found that in stage S1, significantly related genes were enriched mainly in the TCA cycle and the biosynthesis of secondary metabolite pathways. The activity of these pathways leads to the release of CO_2_ and heat in this stage and the synthesis of citrate, trehalose, and succinic acid, which enter stage S2 to participate in the TCA cycle and the biosynthesis of secondary metabolites. In stage S2, significantly related genes were enriched mainly in the biosynthesis of amino acids and the biosynthesis of secondary metabolite pathways. During this stage, CO_2_ is released, and L-proline and L-arginine are synthesized, at which point they enter arginine and proline metabolism and other metabolic pathways. The changes in these three stages corresponded to the increase and decrease in temperature between 12 hours and 48 hours.

## DISCUSSION

At present, the SSF of *Bb* primarily utilizes widely available agricultural byproducts such as wheat bran, soybean powder, and rice. Amino acids, as fundamental substances for biosynthesis, play crucial roles in SSF (30). The metabolism of arginine and proline is closely related to soybean SSF, and arginine treatment significantly increased the desired product during soybean fermentation (31). In the present study, we demonstrated transcriptional and metabolic differences in *Bb* fermentation at different time intervals. Transcriptomic and metabolomic data revealed physiological and biochemical changes in *Bb* during fermentation. High temperatures and CO_2_ concentration limitations hinder the growth of *Bb* (13). Additionally, we discovered for the first time that arginine can influence the SSF of *Bb* and that arginine metabolism is an important metabolic pathway through which this fungus decomposes the culture medium (Fig. 3). Our findings may provide an important research perspective on how *Bb* decomposes and utilizes culture media. The addition of exogenous arginine promoted the growth of *Bb* in the fermentation culture (Fig. 4).

This work reveals new biochemical and genetic mechanisms related to arginine in the SSF of *Bb* and investigates the effects of fermentation conditions on fungi during the fermentation of *Bb*. Protein digestion and absorption are attributed to the microbial utilization of substrates for carbon and energy to produce fungal proteins during SSF (32). The biosynthesis of 12-, 14-, and 16-membered macrolides; carbon metabolism; and the biosynthesis of various alkaloids in SSF provide energy for the fungi, increasing their biological activity (33, 34). Lipids and lipid-like molecules, as well as organic acids and derivatives, are the main secondary metabolites contributing to TCMs in SSF (35, 36). TCGs are enriched primarily in ribosomes and spliceosomes, which are essential metabolic stages for microorganisms in SSF (37).

**Fig 5:**
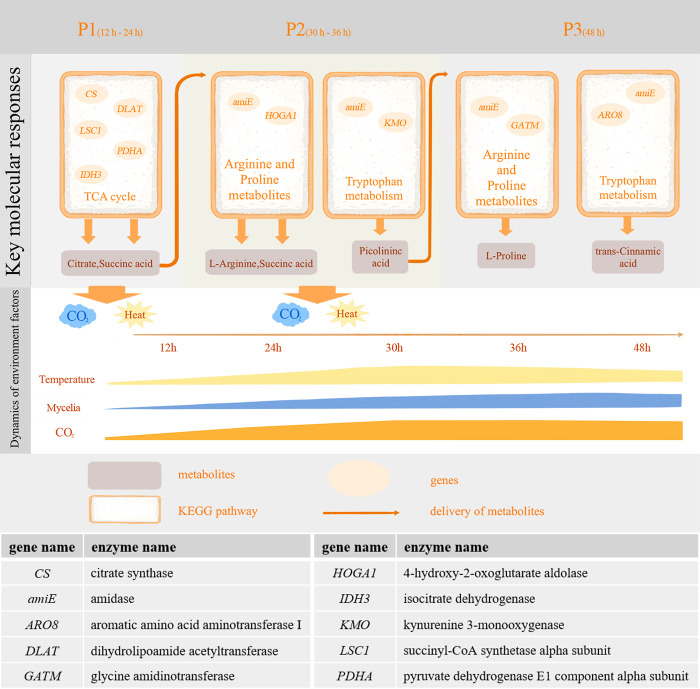
Elucidation of multiomics features during SSF of *Bb* within Stages I–II. The key molecular responses revealed trends in metabolites and genes across the three stages. The lower part shows the dynamics of the environmental factors.

In this study, we speculate that citrate also potentially contributes to promoting hyphal growth during the SSF of *Bb*. Citrate appears in the TCA cycle, and research has shown that supplementation with sodium citrate increases the final yield from SSF, significantly increasing carbon flux and ATP production (38). Metabolite TCMs such as glutamine indirectly synthesize arginine via the arginine metabolism pathway. Metabolites such as succinic acid and L-proline are significantly expressed between 12 hours and 48 hours (Fig. 5), potentially playing crucial roles in fungal SSF. In the future, we will conduct more research to validate the effects of these metabolites on the SSF of *Bb*, aiming to identify substrates more suitable for its SSF.

With respect to temperature-related genes, genes involved in the TCA cycle, such as *CS* (citrate synthase), *DLAT* (dihydrolipoamide acetyltransferase), *LSC1* (succinyl-CoA synthetase alpha subunit), *PDHA* (pyruvate dehydrogenase E1 component alpha subunit), and *IDH3* (isocitrate dehydrogenase), may contribute to the SSF of *Bb*. Studies have explored significant changes in the expression of genes involved in the TCA cycle in wheat bran-based media through transcriptomic and genetic analyses (39). Additionally, the gene *GLUL* (glutamine synthetase) is involved in arginine metabolism and the synthesis of glutamine, indirectly contributing to arginine synthesis. Our subsequent studies will be based on these genes, and we will attempt strain improvement through genetic engineering, which could significantly enhance fermentation processes for *Bb*.

As an emerging biopesticide, *Bb* has the most promising applications on the market. This study explored physiological and biochemical changes during fermentation and provides insights into ways to standardize and optimize SSF. First, heat accumulation is one of the challenging issues in SSF, leading to substrate contamination and limiting strain growth. This study identified arginine as a key component and showed that adding exogenous arginine to the medium can improve thermal issues and successfully increase *Bb* yields. However, the practical application of arginine in production still needs to be studied. We can optimize media formulations in production, replacing the medium’s components with formulations rich in arginine. Nevertheless, the feasibility of this method has not been experimentally validated, necessitating further exploration of its stability and effectiveness. Furthermore, this study explored the mechanisms by which other metabolites and genes influence the SSF of *Bb*, suggesting that other metabolites and genes also have the potential to improve *Bb* production. Modern genetic engineering methods can also contribute to the production of *Bb*. Modifying strains through genetic engineering, such as those identified in this study—the genes *CS* (citrate synthase), *DLAT* (dihydrolipoamide acetyltransferase), *LSC1* (succinyl-CoA synthetase alpha subunit), *PDHA* (pyruvate dehydrogenase E1 component alpha subunit), and *IDH3* (isocitrate dehydrogenase)—could positively impact the production of *Bb*. Through genetic engineering, we may be able to increase the strain’s ability to decompose the culture medium without altering its composition, thus improving fermentation efficiency. This method ensures not only cost-effectiveness but also stability. However, it is crucial to conduct biological assays to verify that genetically modified strains maintain equal potency and yield post-modification. In summary, this research revealed that arginine promotes the growth of *Bb*, mitigating the negative impacts of high temperatures during fermentation. Nevertheless, the feasibility and technical methods of its application in production require further attention and investigation.

## Data Availability

All data are available in the article or as supplemental material. The raw data of the RNA-seq and genome sequencing are openly available in the National Center for Biotechnology Information BioProject under accession numbers PRJNA1192082 and PRJNA1163847. The raw data of the metabolomic analysis can be found in the supplemental material (Table S3). Other data or materials generated in this work are available upon request to the corresponding authors.

## SUPPLEMENTAL MATERIAL

Figure S1 aem.02134-24-s0001.tif Changes in the contents of CO2, temperature, mycelia, and spores during the solid fermentation process of *Bb* within 0 h to 96 h.

Figure S2 aem.02134-24-s0002.tif Temperature-correlated genes of *Bb* solid fermentation medium within Stages I and II.

Figure S3 aem.02134-24-s0003.tif Carbon metabolism.

Figure S4 aem.02134-24-s0004.tif Genetic and metabolic cluster analysis diagram of Stages I and II.

Table S1 aem.02134-24-s0005.xlsx Gene-specific primer pairs.

Table S2 aem.02134-24-s0006.xlsx Correlation analysis.

Table S3 aem.02134-24-s0007.xlsx Raw data of the metabolomic analysis.

ASM does not own the copyrights to Supplemental Material that may be linked to, or accessed through, an article. The authors have granted ASM a non-exclusive, world-wide license to publish the Supplemental Material files. Please contact the corresponding author directly for reuse.
