# Supplementary figures and images for: Arginine accumulation suppresses heat production during fermentation of the biocontrol fungus Beauveria bassiana
Source: Appl Environ Microbiol. 2025 Feb 5;91(3):e02134-24. doi: 10.1128/aem.02134-24 (PMC11921393; doi:10.1128/aem.02134-24)

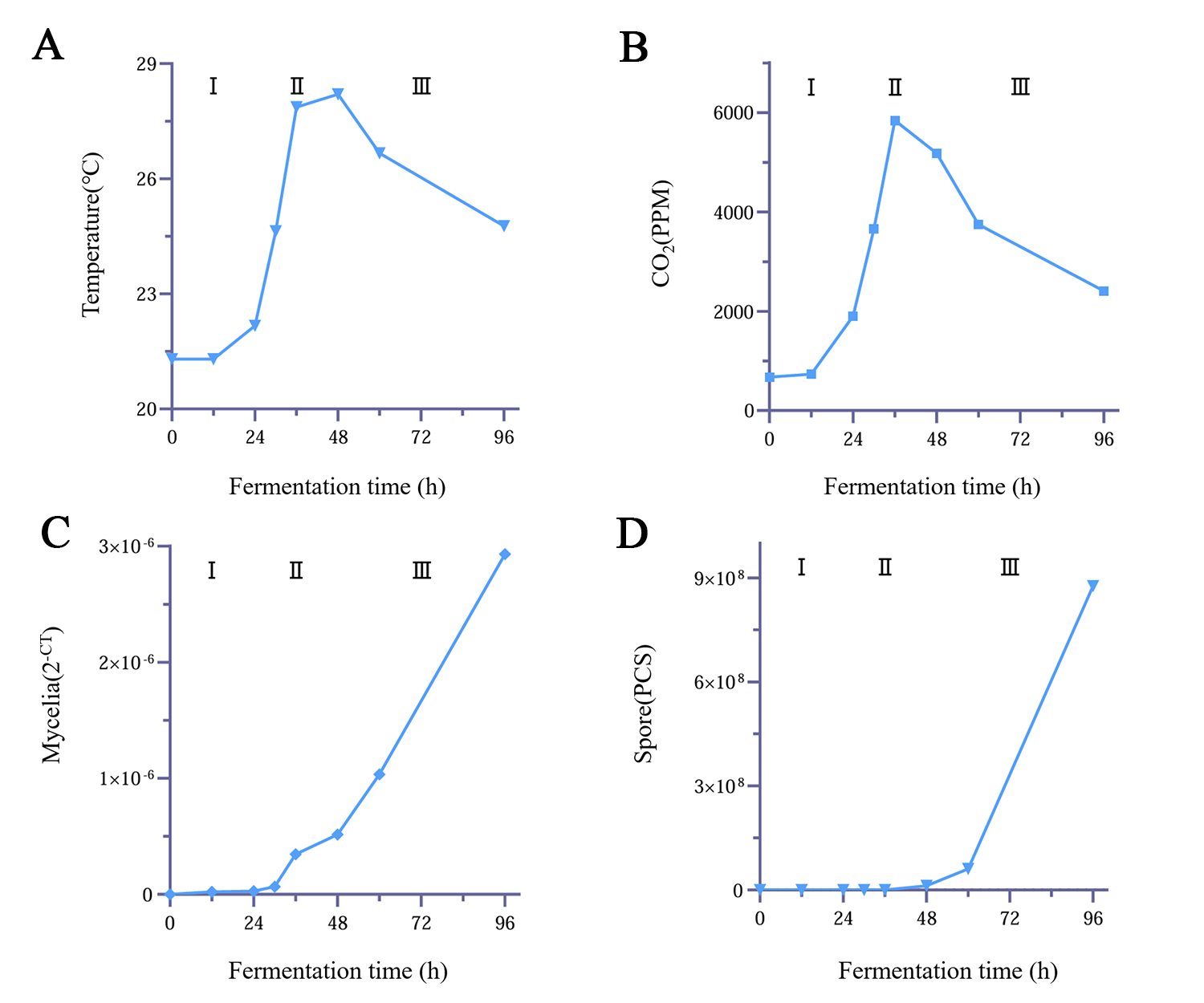

Supplement: Figure S1 — Changes in the contents of CO2, temperature, mycelia, and spores during the solid fermentation process of Bb within 0 h to 96 h. [file aem.02134-24-s0001.tif]

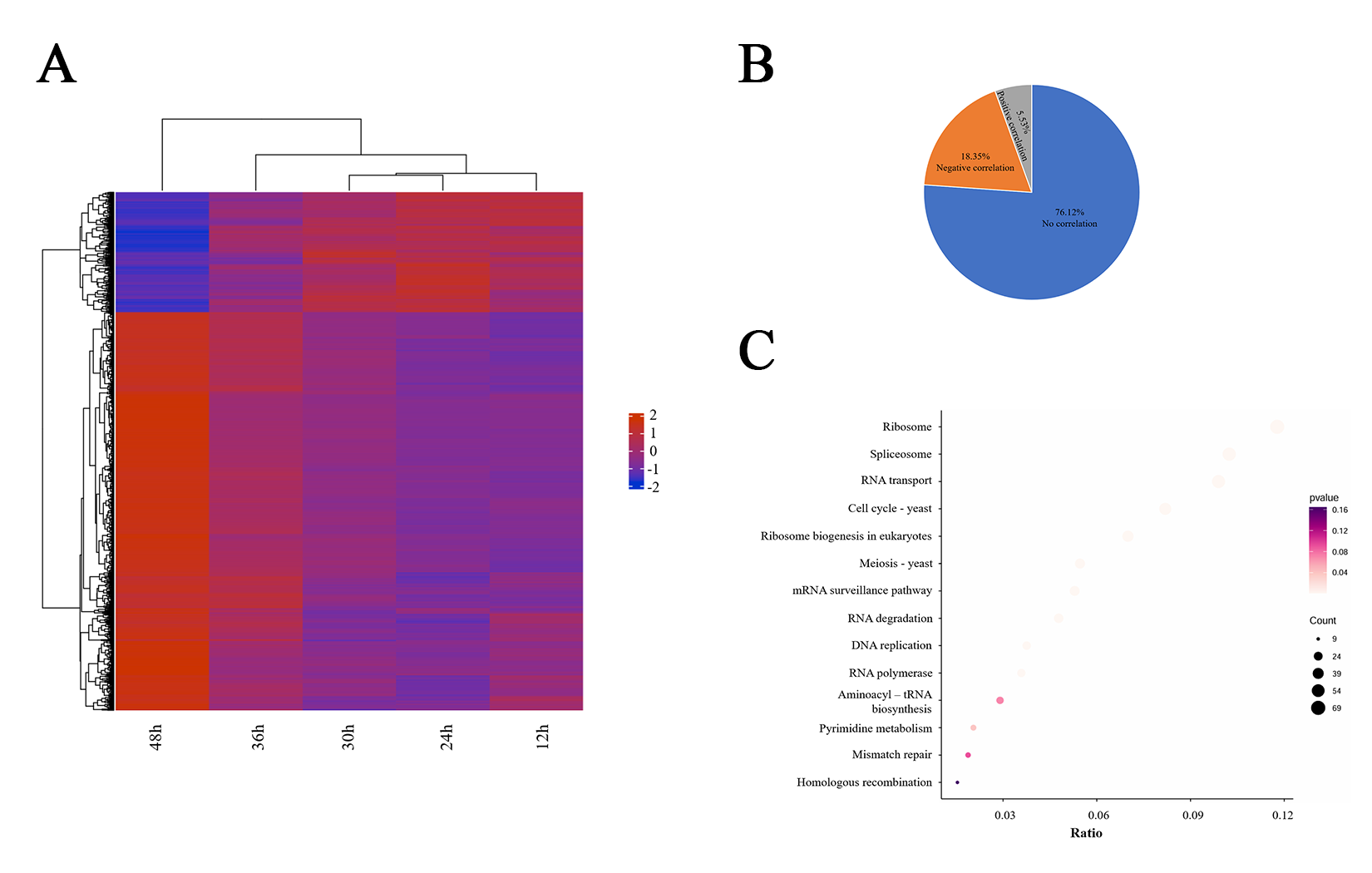

Supplement: Figure S2 — Temperature-correlated genes of Bb solid fermentation medium within Stages I and II. [file aem.02134-24-s0002.tif]

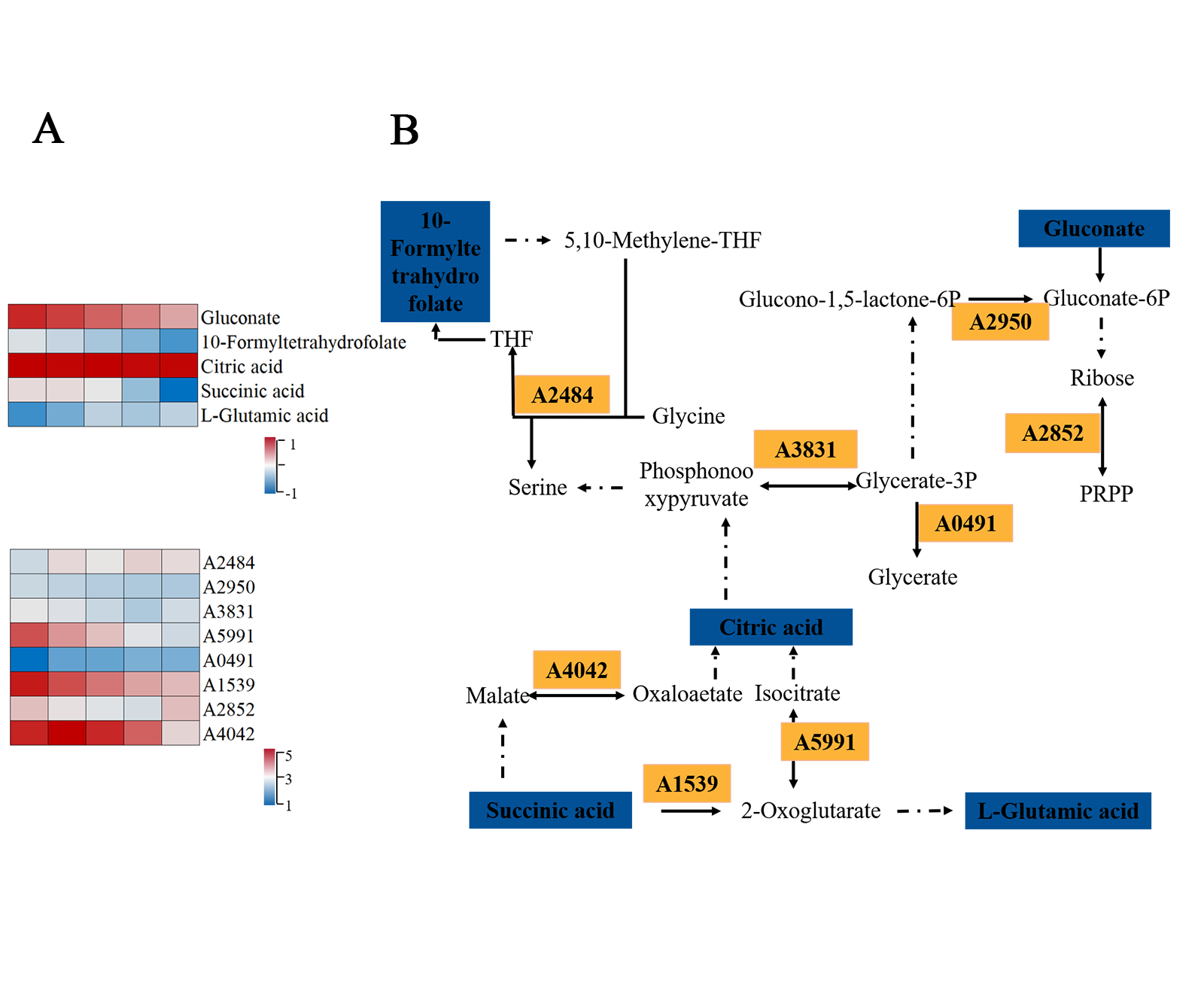

Supplement: Figure S3 — Carbon metabolism. [file aem.02134-24-s0003.tif]

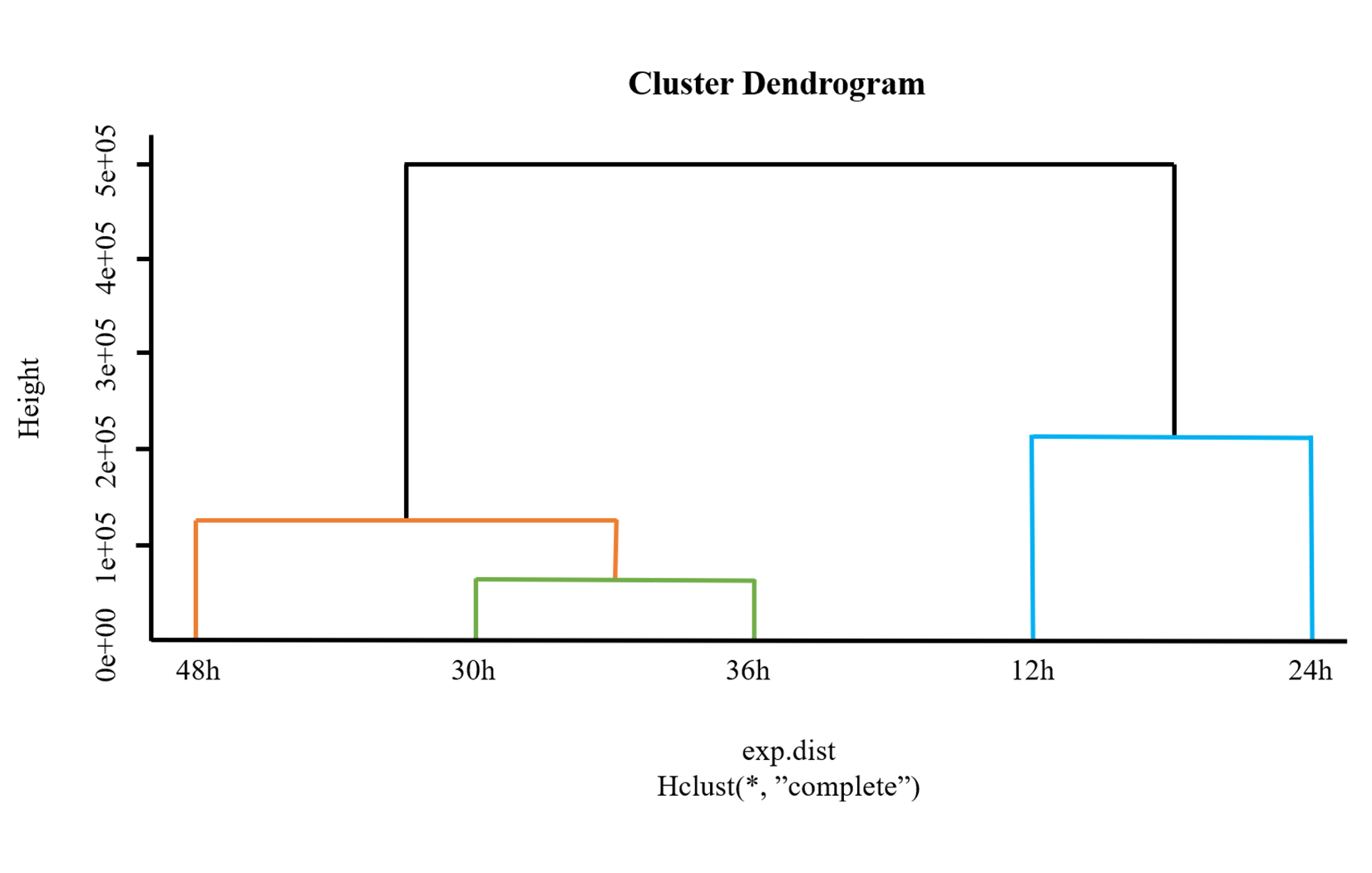

Supplement: Figure S4 — Genetic and metabolic cluster analysis diagram of Stages I and II. [file aem.02134-24-s0004.tif]
